# Toward a more embedded/extended perspective on the cognitive function of gestures

**DOI:** 10.3389/fpsyg.2014.00359

**Published:** 2014-04-24

**Authors:** Wim T. J. L. Pouw, Jacqueline A. de Nooijer, Tamara van Gog, Rolf A. Zwaan, Fred Paas

**Affiliations:** ^1^Department of Social Sciences, Institute of Psychology, Erasmus University RotterdamRotterdam, South Holland, Netherlands; ^2^Early Start Research Institute, University of WollongongWollongong, NSW, Australia

**Keywords:** gestures, embodied cognition, embedded cognition, extended cognition

## Abstract

Gestures are often considered to be demonstrative of the embodied nature of the mind (Hostetter and Alibali, 2008). In this article, we review current theories and research targeted at the intra-cognitive role of gestures. We ask the question how can gestures support internal cognitive processes of the gesturer? We suggest that extant theories are in a sense *disembodied*, because they focus solely on embodiment in terms of the sensorimotor neural precursors of gestures. As a result, current theories on the intra-cognitive role of gestures are lacking in explanatory scope to address how gestures-as-bodily-acts fulfill a cognitive function. On the basis of recent theoretical appeals that focus on the possibly embedded/extended cognitive role of gestures (Clark, 2013), we suggest that gestures are external physical tools of the cognitive system that *replace and support* otherwise solely internal cognitive processes. That is gestures provide the cognitive system with a stable external physical and visual presence that can provide means to think with. We show that there is a considerable amount of overlap between the way the human cognitive system has been found to use its environment, and how gestures are used during cognitive processes. Lastly, we provide several suggestions of how to investigate the embedded/extended perspective of the cognitive function of gestures.

## INTRODUCTION

Gestures reflect internal cognitive processes. This is arguably the most fundamental, uncontroversial, and straightforward assumption in the current literature concerning gesticulation. Gestures provide a “window on the mind” (Goldin-Meadow, 2003), which provides a peek into the “embodied nature of the mind” (Hostetter and Alibali, 2008). In less metaphorical terms, it is argued that gestures are direct outcomes of multimodal, sensorimotor or embodied representations that constitute thought processes and speech production. Although not all theoretical perspectives on the function and underpinnings of gestures suggest a purely sensorimotor based approach to mental representations (see Krauss, 1998; Kita, 2000 for alternative views), it is commonly held that activation of the motor-system supports speech production and thought, at least when the conceptual content is visuospatial in nature (Alibali, 2005). Several perspectives on gesticulation (e.g., McNeill, 1992; Kita, 2000; Wesp et al., 2001) have abandoned the view that gestures are merely communicative tools that are elicited *after* central cognitive processes (e.g., lexical retrieval, conceptualization) have taken place (Graham and Argyle, 1975; Kendon, 1994). Instead, in these perspectives the motor-system has been upgraded from a mere output system to a constitutive system for (some of the) central processes underlying thought and speech production. This resonates well with a wider movement in embodied cognitive science (Wilson, 2002; Shapiro, 2010) in which mental representations are thought to be multimodal (Barsalou, 1999, 2008; Svensson, 2007) and coupled to the body’s current state (Glenberg and Kaschak, 2002).

In this article, we focus on the possible intra-cognitive function of gestures, as opposed to their inter-cognitive or communicative function, which we will touch upon only briefly. That is, gestures seem to support internal cognitive processes of the gesturer (e.g., Rauscher et al., 1996; Goldin-Meadow et al., 2001; Morsella and Krauss, 2004; Marstaller and Burianová, 2013). We argue that the current theoretical “embodied” movement in gesture research has fueled the upsurge of inquiry into the beneficial role of gestures in cognitive processes such as speech and visuospatial cognition, but that this line of thought is underspecified with regard to explaining how gestures *as bodily movements* aid cognitive processing. In a sense, current perspectives on gestures are still *disembodied* and too *internalistic* because they seem to implicitly reduce gestures to *cognitively trivial* bodily outputs of (sensorimotor) neural precursors.

We seek to provide a more embodied account of gesticulation on the basis of recent philosophical and theoretical appeals within embodied cognitive science (e.g., Wilson, 2002) that focus on the possibly embedded/extended role of gestures (Kirsh, 1995; Clark, 2008, 2013; Wheeler, 2013), and a review of related empirical literature (e.g., Gray and Fu, 2004; Kirsh, 2009). This account is “more embodied” because embedded/extended perspectives traditionally seek to provide an anti-internalist perspective on cognition (e.g., Hutchins, 1995a), in which cognition is understood as being on-line, that is, being tightly coupled with, embedded in, if not extended over, the body and the environment (Shapiro, 2010). This stands in stark contrast with more internalist notions of embodiment that are currently dominating the gesture literature and that focus on decoupled, or “off-line” cognition and the sensorimotor nature of mental representations (Wilson, 2002). We suggest that the embedded/extended account of the cognitive function of gestures could be successful in explaining how gestures fulfill a cognitive function if it makes clear how gestures as self-generated bodily acts *generate and support* rather than execute thought processes (Clark, 2013). Therefore, we focus on the idea that gestures may at times serve as external tools of the cognitive system that *replace and support* otherwise solely internal cognitive processes. By reviewing research on the beneficial role of gesture production in (visuo-spatial) cognition (e.g., Chu and Kita, 2008; Delgado et al., 2011) and connecting the resulting insights with research on embedded cognition (e.g., Kirsh and Maglio, 1994; Hutchins, 1995a; Gray and Fu, 2004) we aim to contribute to a more embedded/extended account of gestures.

Before we will elaborate on the main goals of this paper, we need to point out what this article is not about. First, we do not suggest that current perspectives in the gesture literature are incorrect. In fact, our embedded/extended perspective is largely complementary to, and in some instances builds on, contemporary accounts of the function of gestures we review here. Second, although we argue in favor of a more embodied account of gestures and their cognitive function, this does not require us to make any additional, more radical, claims about the supposed sensorimotor nature of conceptual representations that are currently under discussion in the literature (e.g., Dove, 2010; Arbib et al., 2014; Zwaan, in press). Third, we will not provide philosophical claims about whether gestures should be considered as an extended as opposed to an embedded cognitive phenomenon (e.g., Adams and Aizawa, 2001; Clark, 2008, 2013; Wheeler, 2013). That is, we do not make explicit claims about whether gestures as extra-neural events are part of the cognitive process (extended claim) or whether gestures merely support internal cognitive processes but strictly speaking should not be considered as part of the cognitive process (embedded claim). Rather, we aim to provide an empirical view through the embedded/extended perspective, on the basis of the shared anti-internalist goal of these perspectives, by focusing on extra-neural factors that support, shape, and replace internal cognitive processes. We suggest that our embedded/extended account of the cognitive function of gestures can fill an explanatory gap in the current literature concerning the possible intra-cognitive role of gestures and is supported by extant findings.

This article is structured into four main sections. The next section reviews findings that show that co-speech and -thought gestures have a (beneficial) cognitive function (primarily in visuospatial cognition). Section three provides an overview of some important theoretical perspectives on the role of gestures in cognition. We suggest that the current theoretical perspectives on the function and underpinnings of gestures leave an explanatory gap concerning how gestures as external bodily acts might be conducive to internal cognitive processes. Having exposed the explanatory gap, we introduce an embedded/extended account of gestures (Clark, 2008, 2013) and provide a new interpretation of the research reviewed in the previous section in light of recent research in the field of embedded cognition (Kirsh and Maglio, 1994; Ballard et al., 1995; Gray and Fu, 2004; Kirsh, 2009; Risko et al., 2013). Finally, we summarize and discuss our main points.

## THE FUNCTION OF GESTURE: EMPIRICAL EVIDENCE

### THE INTER-COGNITIVE ROLE OF GESTURES

Before we consider evidence for the beneficial or supportive role of gestures for cognitive processes, it is important to acknowledge the evidence for the common assertion that gestures fulfill a communicative function. When speakers produce gestures, this seems to be intended to increase listeners’ understanding of their message. Indeed, when speaker and listener are face-to-face, more gestures with semantic content are produced than when there is no visual contact (Alibali et al., 2001). Also, when speakers are aware of listeners’ knowledge gaps, they tend to convey the information unknown to listeners in both speech and gesture, while they tend to only use verbal information when relevant knowledge is already shared between the interlocutors (Holler and Stevens, 2007). These results suggest that speakers adjust their gestures for their listeners’ benefit. And indeed, listeners’ comprehension has been shown to improve by speakers’ use of gestures from an early age on. For example, 3- to 5-year-olds understand indirect requests (Kelly, 2001) and new abstract concepts (Valenzeno et al., 2003) better when the request is accompanied by deictic (i.e., pointing) gestures. In addition, preschoolers understand complex spoken messages better when these are accompanied by representational gestures (McNeil et al., 2000). Moreover, co-speech gestures do not only contribute to *what* is understood, but also to *how* something is understood. When deictic gestures are used, listeners are more likely to correctly interpret utterances compared to when the utterance was not combined with a gesture, suggesting that co-speech gestures play a role in pragmatic understanding. For example, when hearing the utterance “it’s getting hot in here,” people were sooner inclined to interpret this as an indirect request (i.e., could you please open the window) when the speaker pointed to the window, than when the speaker did not point, in which case the listener might interpret the utterance as a mere statement (Kelly et al., 1999). All in all, there is a great deal of evidence for the contention that gestures fulfill inter-cognitive (i.e., communicative) functions (Goldin-Meadow and Alibali, 2012).

### THE INTRA-COGNITIVE ROLE OF GESTURES

There is mounting evidence that gestures fulfill intra-cognitive functions in addition to inter-cognitive ones. This is relevant to our present purposes. For example, co-speech gestures affect speakers’ own cognitive processes. Several studies have suggested that lexical access is disrupted or promoted when gesticulation is prohibited vs. allowed to naturally emerge. When speakers are prohibited from gesturing during speech with spatial content, they are less fluent than when gesticulation is allowed, suggesting that lexical access is disrupted (Rauscher et al., 1996; Morsella and Krauss, 2004; see, however, Hoetjes et al., 2014). Moreover, speech is more fluent when co-speech gestures are produced and gesture rates are higher when lexical access is difficult (e.g., during the tip of the tongue phenomenon; Chawla and Krauss, 1994). Furthermore, when gesticulation is prohibited, the content of speech is less likely to be spatial in nature, suggesting that gestures support speech that is spatial in content (Rimé et al., 1984). Not only can online speech be influenced by co-speech gestures, these gestures can also have an influence off-line. For example, making gestures during the recollection of a previous event, can improve retrieval of details of that event compared to when gesticulation is not allowed (Stevanoni and Salmon, 2005). In addition, gesticulation prior to recalling previously learned words aids recall performance (De Nooijer et al., 2013).

Gestures primarily arise during the processing of visuospatial information (e.g., Alibali et al., 2001; Seyfeddinipur and Kita, 2001; Allen, 2003; Kita and Özyürek, 2003). For example, people are more likely to gesture when describing visual objects from memory as opposed to when the object is visually present (Wesp et al., 2001; Morsella and Krauss, 2004; see also Ping and Goldin-Meadow, 2010), although gesticulation also occurs when the object is present (Morsella and Krauss, 2004). Moreover, gestures occur more often when objects are difficult to describe in speech, such as complex, not easily describable drawings (Morsella and Krauss, 2004). Indeed, the emergence of gesticulation appears to be related to the cognitive demands of the task (Goldin-Meadow et al., 2001; Wagner et al., 2004; Ping and Goldin-Meadow, 2010; Cook et al., 2012; Marstaller and Burianová, 2013; Smithson and Nicoladis, 2014). For example, participants who were given the dual task of remembering letters while explaining a difficult math problem, remembered more letters when they were allowed to gesture while explaining the problem than when they were not allowed to gesture (Goldin-Meadow et al., 2001). This suggests that gesticulation reduced the working memory load imposed by explaining the math problem, leaving more capacity available for performing the secondary task of remembering letters. Gesticulation when describing a mental rotation problem emerges primarily when describing the task-relevant rotation itself as opposed to describing the task-relevant static end-point of the rotation (Hostetter et al., 2011). This finding suggests that it is the high spatial cognitive demand, which is arguably higher during dynamic spatio-temporal rotation as opposed to describing static spatial information, that invokes the use of gestures (see also Smithson and Nicoladis, 2014). Furthermore, it has been found that encouraging participants to gesture during a mental rotation task enhances their performance (Chu and Kita, 2011).

The findings described here primarily involved iconic gestures. However, even deictic (pointing) gestures occur more often when cognitive demand is higher. Infants and young children (between 1 and 2 years of age) sometimes point for non-communicative reasons (Bates et al., 1975; Delgado et al., 2009). Furthermore, pointing gestures can aid the regulation of the speaker’s attention in non-communicative and challenging problem-solving situations (Delgado et al., 2011). In two studies, children ranging in age from 2 to 4 years old saw a toy being hidden in one of three containers on a rotation table. This was followed by a delay of 45–60 s during which the children either had to remember where the toy was hidden by the experimenter (cognitive demand group) or had to wait for the experimenter to retrieve the toy for them. During the delay the experimenter left the room. Additionally, the difficulty of the memory task was varied for half of the trials such that the table was turned for 540°. Analysis of the video-taped sessions showed not only that solitary pointing gestures occurred, but also that they occurred significantly more often in the cognitive demand condition than in the waiting condition (although no effects were found for task difficulty). A second experiment with children ranging from 4 to 6 years old who performed a picture-matching task showed that constraining gestures resulted in poorer performance on the task than non-constraining gestures, but only for children who habitually pointed in the constrained condition, suggesting a cognitively beneficial role of solitary pointing gestures. This finding is surprising because deictic gestures have primarily been considered as serving communicative functions (Tomasello et al., 2007). Additional research on pointing gestures was conducted in the context of keeping track of counting. Children, adults, and even primates effectively use the hands in counting objects by pointing and touching gestures as to mark counted objects, and synchronize with counting expressed in speech (Boysen et al., 1995; Kirsh, 1995; Alibali and DiRusso, 1999). For example, participants who were allowed to use their hands for pointing during the counting of coins were faster and made fewer mistakes than those who were not allowed to use their hands (Kirsh, 1995). Thus, pointing gestures sometimes regulate visuo-spatial attentional processes, being especially helpful under high cognitive task demands.

These results converge with a recent correlational study that examined whether individual differences in spatial working memory capacity, spatial transformation ability, and conceptualization ability (amongst others) were associated with frequency of use of several types of gestures (Chu et al., 2013). Lower scores on all of these variables predicted higher frequency of spontaneously produced representational and conduit^1^ gestures in a natural setting. Other evidence is consistent with this pattern. Particularly people with low working memory capacity are negatively impacted on a working memory task when they are not allowed to gesture as opposed to people with high working memory capacity (Marstaller and Burianová, 2013). Thus, in addition to the findings that gestures emerge during spatial information processing, gestures are also more likely to be produced by, and more likely to affect cognitive processes of, people with low spatial working memory and information processing ability (see also Chu and Kita, 2011).

Further evidence for gesturing as a compensatory mechanism comes from a study by Chu and Kita (2008). The type of spontaneous gestures that participants used during a mental rotation task followed a trajectory from external to more internalized solution strategies. That is, participants first gestured concretely *as if* manipulating the object to be rotated and subsequently changed their strategy and used their flat hand as stand-in for the object that needed to be rotated. Moreover, frequency of gesture use in aiding a spatial rotation task diminished over time, suggesting that cognitive operations became gradually internalized. A related phenomenon is that intermediate advanced abacus users use gestures during mental calculation. In the absence of the abacus, trained participants apply finger gestures as if manipulating an abacus ready to hand; but as abacus users become more advanced, they exhibit a reduced reliance on gestures during mental calculation (Hatano et al., 1977; Hatano and Osawa, 1983). In line with the findings of Chu and Kita (2008) this shows that the use of gestures becomes more infrequent as familiarity with the task increases. Moreover, when describing the solution of a particular spatial problem, people’s gesticulation aligns with the medium that the problem has been introduced in Cook and Tanenhaus (2009). For example, participants who described solutions of the Tower of Hanoi with physical disks as opposed to a computer simulation tended to spontaneously produce gestures that aligned with the physical actions performed with physical disks.

Thus, if we consider (a) that working memory capacity is limited, and (b) that new tasks often impose a higher working memory demand that diminishes as the learner becomes more experienced with a task (e.g., Chase and Ericsson, 1982; Kalyuga et al., 2003) then the findings we just reviewed suggest that gestures are likely to emerge in novel situations so as to provide the cognizer with some kind of external support. We will discuss the nature of this external support in our embedded/extended account of the cognitive function of gestures.

Finally, gestures can aid in acquiring a solution during problem solving (Alibali et al., 2004; Stephen et al., 2009; Boncoddo et al., 2010). For example, participants were presented with two glasses with differing widths and equal heights and were asked to imagine the glasses being filled with water to the same level. Participants judged whether the water would spill when glasses were rotated at equal angles (Schwartz and Black, 1999). Participants were able to predict the answer correctly much more often when rotating the empty glasses with their eyes closed, compared to when they were only allowed to think about the solution (i.e., mentally rotate). Although the previous study was in a sense a form of direct action (by allowing the objects to be manipulated), there is evidence that suggests that gestures, as non-direct manipulations, equally support the use of particular problem-solving strategies. For example, a study in which participants were presented with an interlocking gear problem (Alibali et al., 2004) found that they judged the direction of movement of a gear through different strategies, depending on whether or not gesticulation was allowed. When they were allowed to gesture, participants were more likely to simulate the rotations of each gear by finger gestures in order to provide the solution of the end-gear’s rotational direction (depictive strategy), whereas participants who were prohibited from gesticulation were more likely to achieve the solution through the parity rule (direction gear *x* has the same direction as gear *x* + 2). Note that the participants who used the depictive strategy were not better at the task than those using the parity rule (Alibali et al., 2004; also see Hegarty et al., 2005). Indeed, the parity rule strategy is generally considered to be the most effective strategy (Boncoddo et al., 2010). It is interesting in this regard to note that preschoolers are more likely to achieve understanding of the parity rule through gesticulation (Boncoddo et al., 2010). That is, preschoolers who used more gestures supporting a depictive strategy, more efficiently acquired a strategy based on the parity principle, in comparison to preschoolers who gestured less. Thus in this particular instance, the repeated use of gestures by participants is more likely to lead to discovery of new strategies during problem-solving although the use of gestures does not necessarily invite learners to adopt the most efficient strategy (see also Stephen et al., 2009).

The research reviewed here provides evidence that gestures have an intra-cognitive cognitive function for the gesturer. Furthermore, it produces two intriguing and related questions that we think need to be answered in a theoretical account of the cognitive function of gesticulation. First, why do gestures occur more often when cognitive demand is high? Second, why are spatial cognitive ability and working-memory capacity negatively related to the use of gestures?

## CURRENT THEORY ABOUT THE ORIGIN AND FUNCTION OF GESTURE

In this section, we will discuss several prominent accounts that aim to elucidate the underlying mechanisms and function of gestures, most prominently the Gesture-as-Simulated-Action account (GSA; Hostetter and Alibali, 2008) and subsequently the Lexical Gesture Process (LGP) model (Krauss et al., 2000), the Information Packaging Hypothesis (IPH; Kita, 2000), and the Image Maintenance Theory (IMT; Wesp et al., 2001). We evaluate these models directly after summarizing their main points, by assessing their explanatory power regarding the question: how do gestures-as-bodily-acts support cognitive processes?

We have chosen to address this collection of accounts for several reasons. The GSA account is a prominent contemporary account that attempts to integrate the literature of embodied cognition and the literature on gesture into a single perspective. Yet, as mentioned in the introduction, it seems that this attempt has resulted in a “disembodied” perspective on gesticulation. The other accounts have been very influential in elucidating the cognitive function of gestures. Moreover, they differ significantly from the GSA account but also from each other. The result is a representative (but not exhaustive) overview of theories about the possible cognitive function of gestures.

### GESTURE-AS-SIMULATED-ACTION ACCOUNT

The GSA account (Hostetter and Alibali, 2008) relies heavily on the insights from embodied cognition that representations are based on the sensorimotor system (Barsalou, 1999, 2008; Glenberg and Kaschak, 2002). This embodied view is supported by mounting evidence that perceptuo-motor faculties of the brain are activated during concrete but also supposedly symbolic and abstract conceptual processes (e.g., Barsalou, 2008; Pulvermüller et al., 2014). For example, merely reading words that have olfactory, gustatory, or motor connotations (e.g., garlic, jasmine, salt, sour, kick, pick) as opposed to reading neutral words, activates brain regions that are involved in smelling, tasting, and moving (Hauk et al., 2004; Gonzalez et al., 2006; Barrós-Loscertales et al., 2012).

The GSA approach predicts that cognitive processes, such as conceptual processing, co-occur with sensorimotor reactivations. More importantly it is contended that meaningful cognitive processing is *dependent* on these reactivations or simulations of sensorimotor states (Barsalou, 2008; Hostetter and Alibali, 2008). Indeed, conceptual processing is hampered when participants are primed with inconsistent perceptual or motor information (e.g., Glenberg et al., 2005; Kaschak et al., 2006). For example, participants are quicker in verifying the sensibility of sentences (such as “Andy delivered the pizza to you vs. you delivered the pizza to Andy”) when their response actions were consistent with the implied motion of the sentences (moving the hand forward or backward), whereas they were slower when the movement contrasted with the implied motion (Glenberg and Kaschak, 2002). As such, it is suggested that induced sensorimotor states impinge on conceptual representational states since both systems are tightly coupled (Barsalou, 2008).

Hostetter and Alibali (2008) have suggested that the phenomenon of co-speech and co-thought gestures fits nicely with the idea that cognitive processing depends on activations in the sensorimotor system. In fact, according to the GSA account gestures *are* the bodily realizations (or as they call it, “visible embodiments”) of otherwise covert sensorimotor activations. The main question that the GSA account aims to address, therefore, is how sensorimotor activations come to be reflected in gestures. Hostetter and Alibali (2008, p. 503) first provide a simple answer: “Simulation involves premotor action states; this activation has the potential to spread to motor areas and to be realized as overt action. When this spreading activation occurs, a gesture is born.” More specifically, the GSA account suggests that gestures emerge through sensorimotor re-activations underlying thought and speech processing that “leak into” the motor-executive system:

“As an analogy, we might imagine activation spreading from premotor areas to motor areas through a gate. Once the gate is opened to allow more activation for one task (speaking), it may be difficult to inhibit other premotor activation (that which supports gestures) from also spreading through the gate to motor areas, the activation for the simulations ‘rides along’ and may be manifested as a gesture” (Hostetter and Alibali, 2008, p. 505).

Hostetter and Alibali (2008) further propose three underlying factors that determine when gestures are likely to occur. First, the strength of the particular perceptuo-motor activation must surpass a certain *gesture threshold* for actual physical embodiment (i.e., gesticulation) to arise. This activation strength is dependent on the degree to which speakers evoke visuospatial imagery during conceptual processing. For instance, they argue that the same conceptual content can be processed verbal-propositionally or with visuo-spatial imagery (e.g., in the case of route-descriptions), the latter type of encoding being more likely to evoke gesticulation (e.g., Alibali et al., 2001; Seyfeddinipur and Kita, 2001; Allen, 2003; Kita and Özyürek, 2003). Second, visuo-motor simulations are likely to evoke gesticulation when the conceptual content that is being processed involves an action. For example, talking about action is likely to evoke gestures because it is dependent on motor-information (Hostetter and Alibali, 2008). Third, it is speculated that the height of speakers’ gesture-threshold can vary across individuals and situations. To illustrate, a higher degree of neural interconnectivity between pre-motor and motor areas may lower the gesture threshold of a particular individual. Furthermore, inhibiting gesticulation requires cognitive effort and as such the threshold might be lowered when cognitive load is high (e.g., Goldin-Meadow et al., 2001).

#### Explanatory power of the GSA account

So how does the GSA account answer our question of how gestures-as-bodily-acts support cognitive processes? First, it is held that speech production and thought processes are dependent on the conceptual system recruiting sensorimotor representations. Furthermore, according to Hostetter and Alibali (2008), gestures arise from and are dependent on the strength of sensorimotor activations. However, the model does not allow the conclusion that gestures-as-bodily-acts aid cognition, because gestures only *execute* sensorimotor information, they do not *produce* it. The sensorimotor information that is produced (e.g., proprioceptive and visual consequences of movement) does not fulfill a cognitive function in the GSA account. This is indicated by the motor-leakage metaphor, as gestures simply “ride along” with sensorimotor activations (Hostetter and Alibali, 2008, p. 505) and can be understood as a mere “outgrowth” (Risko et al., 2013) or “visible embodiments” (Hostetter and Alibali, 2008) of internal embodied simulations. Thus, the GSA account leaves us with the question why do cognitive processes sometimes recruit the body (gestures), as opposed to relying on purely internal mechanisms? Furthermore, what is the explanatory power of the GSA account in terms of the empirical literature on the cognitive function of gestures provided above? Most notably, why is high cognitive demand result in more use of gestures. This is explained by the GSA account in “that inhibiting activation from spreading to a gesture requires more cognitive resources than does producing the gesture” (Hostetter and Alibali, 2008, p. 505). From this point of view, gesticulation is the default and is simply hard-wired with cognitive processes. By accepting this, we would simply deflate the idea of there being any function of gestures as bodily acts, endow the cognitive system with functionally unnecessary expenditure of energy (hand-movements), and allow only a negative cognitive effect of not gesturing. Although this idea of costly active inhibition may very well be a correct explanation for some instances of gesticulation, we think its possible scope for explaining the function of gesture is somewhat reduced by the realization that possessing a superfluous and energy-demanding gesture system does not seem very adaptive or flexible. Moreover, we think that a non-deflationary account of the function of gesture is possible and in fact more promising for understanding the empirical findings on the cognitive function of gestures reviewed in this paper.

### LEXICAL GESTURE PROCESS MODEL

The LGP model proposed by Krauss et al. (2000) tries to explain why speech might be facilitated by gesticulation. According to this theory, gestures do not only fulfill a communicative role, but may serve to facilitate lexical retrieval on the part of the gesturer as well. Gestures that share features with the lexical semantic content of the word will facilitate lexical access. Krauss et al. (2000) hypothesize that this is the case because gesturing results in “cross-modal priming” in which features of the concept represented by the gesture can facilitate lexical retrieval. According to this LGP account, gesture production draws upon the activated representations in working memory that are expressed in speech. The assumption is that the content of conceptual memory is encoded in multiple ways, and that activation of one representational format can spread to activation in another representational format. In this account, gestures derive from non-propositional representational formats (mostly visuo-spatial), as opposed to speech, which draws on propositional symbolic formats. LGP further suggests that non-propositional information becomes expressed in speech through a spatial/dynamic feature selector that transforms spatially and dynamically formatted information into a set of “abstract properties of movement.” The abstract specifications are then translated into a motor program by a motor planner. Motor systems output the set of instructions from the motor planner and the gestural movement is monitored kinesthetically. The motoric features that are picked up by the kinesthetic monitor promote retrieval of the concept for speech through *cross-modal priming*. Krauss and Hadar (1999, p. 21) specify:

“The spatio-dynamic information the gesture encodes is fed via the kinesic monitor to the formulator, where it facilitates lexical retrieval. Facilitation is achieved through cross-modal priming, in which gesturally represented features of the concept in memory participate in lexical retrieval. Of course, it is possible to locate the site of gestural input more precisely (e.g., the grammatical encoder or the phonological encoder).”

#### Explanatory power Lexical Gesture Process model

Does LGP allow for a cognitive role of gestures-as-bodily-acts? That is, does it answer the question why gestures are produced, and how they are cognitively relevant? An affirmative response is appropriate, although the mechanism seems underspecified and unparsimonious. Indeed, when a gesture is outputted by the motor-system, the “kinesthetic” feedback that is produced acts as input to the formulator (i.e., the grammatical or phonological encoder or both) and can then facilitate lexical selection by way of additional cues or “cross-modal priming.” Thus, in this model, motor-information is externalized and is fed back into the system to promote lexical retrieval through supporting the processes of the “grammatical encoder” and the “phonological encoder.” Yet the question remains why this motor-information needs to loop out of the brain and then be retrieved again by the kinesthetic monitor. According to LGP, gesture will only facilitate lexical access when the gesture features match the lexical semantic content of the concept. Therefore, gestures will only facilitate lexical access when the kinesthetic information that was already present in a verbal form is fed back into the formulator. Thus it seems that the brain is “primed” with information that is already present in the internal system, given that gestures are outputs of an already constructed motor program. Thus, it is unclear with what kind of information the cognitive system is primed. Of course, gestures might indeed fulfill this function, but the model currently presented is not very illuminating why and how gestures-as-bodily-acts fulfill a cognitive function. So, although LGP also suggests an intra-cognitive role for gestures, it is still difficult to appreciate the added value of the kinesthetic information that is fed back into the system with regard to cognitive processing.

### INFORMATION PACKAGING HYPOTHESIS

A third prominent theory in the gesture literature is the IPH (Kita, 2000). This theory proposes that gestures aid speech production by breaking images into smaller bits to enhance the verbalize-ability of communicative content. A key idea is that there are two modes of thinking that tend to converge during the linguistic act. There is analytical thinking as opposed to spatio-motoric thinking from which gestures follow, which involves the organization of information through hierarchical structuring and involves decontextualized conceptual templates. According to Kita, these templates can be non-linguistic (in the case of scripts), or linguistic, such as in the case of a lexical item’s semantic and pragmatic specifications. The templates are not multimodal as in the case of the GSA account, thus they do not involve “activation of ‘peripheral’ modules” (Kita, 2000, p. 164), yet can be translated into the other mode of thinking, which is spatio-motoric thinking. The spatio-motoric mode of thinking constitutes gestures and involves information organized in action schemas. Gestures should be considered as actions in a virtual environment, and are derived from practical actions.

A core idea behind IPH is that the two modes of thinking collaboratively organize information during speaking. Kita (2000, p. 163) suggests that (a) “The production of the representational gesture helps speakers organize rich spatiotemporal information”, (b) “Spatio-motoric thinking, which underlies representational gestures helps speaking by providing an alternative informational organization that is not readily accessible to analytic thinking” and (c) “Spatio-motoric thinking and analytic thinking have ready access to different sets of informational organizations. However, in the course of speech production, the representations in the two modes of thinking are coordinated and tend to converge.”

#### Explanatory power Information Packaging Hypothesis

Does IPH have explanatory power of how gestures-as-bodily-acts support cognitive processes? The IPH does not provide a clear account of how gestures aid the “packaging of information” given that gestures are considered as the result of spatio-motoric thinking that is already internally realized. That is, just like the GSA, the IPH seems to regard gestures as mere output of spatio-motoric thinking, with the latter having the actual cognitive function (information packaging). Even if we allow for a possible different reading of the IPH, in which gesticulation actually supports spatio-motoric thinking, the IPH account does not go into any detail about how gestures-as-bodily-acts feedback to or support internal cognitive processes to perform the function of spatio-motoric information packaging.

### IMAGE MAINTENANCE THEORY

The final theory under review here is the IMT by Wesp et al. (2001). Although this theory is only briefly presented in an empirical paper it has become an influential view on the cognitive role of gestures (Alibali, 2005). Arguably, the main thesis of the IMT, which is often contrasted with the LGP, is “that gestures are not directly involved in the search for words; rather, they keep the non-lexical concept in memory during the lexical search, a process of data maintenance not unlike that needed in other problem-solving activities” (Wesp et al., 2001, p. 592). This is further explained; “a prelinguistic representation of spatial information is established through spatial imagery and maintenance of these spatial images is facilitated by gestures” (Wesp et al., 2001, p. 595). Wesp et al. (2001) base this idea on the idea that spatial information is held in the visuospatial scratchpad of working memory (Baddeley, 2003). The items (visuospatial information) in the scratchpad decay rapidly and must be rehearsed to be maintained in working memory. Just like articulatory loops, gestures serve the function of “refreshing” the visual scratchpad to sustain activation of the image in working memory. Importantly, gestures are therefore not necessary for lexical retrieval but may indirectly facilitate it through, “motoric refreshing” of the image (p. 597).

#### Explanatory power Image Maintenance Theory

Does the IMT have explanatory power of how gestures-as-bodily-acts, support cognitive processes? The answer is yes, although much is still needed to understand its function. “Yes” because the IMT suggests that the production of a *physical* gesture supports the maintenance of an internal spatial image (a cognitive process); without the physical gesture the internal spatial image becomes unstable and its activation is likely to decay. Yet, Wesp et al.’s (2001) account does not provide sufficient detail beyond this notion. How do gestures refresh motoric spatial images? What is the mechanism by which gestures-as-bodily-acts refresh motor spatial images? Furthermore, are not gestures redundant given that they provide the gesturer with information that is already present in the system that outputs the gestures (e.g., visual information)? Although these questions remain unanswered, of all the accounts presented here, the IMT is most compatible with an embedded/extended account that assumes gestures are cognitively relevant because they are bodily.

### SUMMARY OF FINDINGS FROM THE THEORETICAL OVERVIEW

In the previous subsections, we have discussed four models that have been put forth to explain the underlying mechanisms of gestures. We sought an answer to our question: how do gestures-as-bodily-acts support cognitive processes? Our review of the literature suggests that the cognitive function of gestures-as-bodily-acts cannot be adequately explained, or remains underspecified, in several different theories about the underpinnings and functions of gestures. In the GSA account gestures are seen as by-products of sensorimotor activation but cease to be supporting cognition the moment they are outputted by the motor-system. The IPH suggests that gestures help package the spatio-motoric thinking during speech, yet this account also assumes that gestures are the result of these processes as they are the realizations of spatio-motoric internal processes; they are pre-packaged the moment they are externalized as gestures and do no packaging of their own. In the LGP account, the gestures that are produced are fed back into the cognitive system to provide it with cross-modal primes. As such, gestures, as physical acts, attain a function. Yet, the LGP account is unclear about what exactly is primed, or what novel information gestures provide to the system, that was not already activated or present. Interestingly, the IMT does seem to ascribe a definite cognitive function to gestures by positing that they support the maintenance of mental images.

It is important to stress that our review is aimed at answering a specific question that may be different from the questions that the theories we discussed were designed to address. We have only considered these theories’ explanations (*explanantia*) of a particular aspect of gesticulation that we think needs to be explained (*explanandum)*, namely how gestures-as-bodily-actions have a cognitive function. This means that we do not suggest that the theories under discussion are wrong, nor do we suggest that they are incompatible with the upcoming perspective; rather the *explanantia* they offer are not (yet) suitable to cover the *explanandum* that is the focus of the current paper. In the next section, we aim to fill this explanatory gap through a more embedded/extended perspective on the cognitive function on gestures.

## TOWARD A MORE EMBEDDED/EXTENDED PERSPECTIVE TO THE COGNITIVE FUNCTION OF GESTURES

In this section, we attempt to answer the main question of how gestures can fulfill cognitive functions. In the following subsection, we will briefly introduce the embedded/extended cognition perspective (inspired by Clark, 2013), which is followed by a representative overview of research in this domain. Subsequently we apply the relevant theoretical and empirical findings to the cognitive function of gestures, which yields challenges and hypotheses for future research.

### AN EMBEDDED/EXTENDED PERSPECTIVE: THEORY AND RESEARCH

Embedded/extended cognition is considered part of the broader development of embodied cognitive science (Wilson, 2002; Shapiro, 2010) and has its roots (amongst others; Gallagher, 2009) in situated cognition (Bredo, 1994), robotics (Brooks, 1991) and the dynamical systems approach to cognition (Chemero, 2009). According to a loose description of “the” embedded/extended perspective on cognition (cf. Wilson, 2002), the main thesis is that the cognitive system is a coupled brain–body–world system (Wheeler, 2007; Clark, 2008). As such, cognition involves an ongoing transaction between current states of the brain, body, and the environment (Clark, 2008). Within this view, the classic internalist picture of cognition is disputed; thinking is something we do, rather than something that simply happens within us. Understanding cognition, therefore, requires a broader level of analysis that allows the study of how we use our body and the world during the unfolding of cognitive processes. For example, Hutchins (1995b) analyzed the goings-on of commercial airlines and suggested that a purely internalist perspective was ill-suited to understand its workings; flying a plane involves task-relevant information that is neither fully instantiated in the cockpit, the pilot, or co-pilots, it is rather distributed among them and all parts work together (see also Hutchins, 1995a). Everyday examples of embedded/extended cognitive phenomena would be, for instance, asking another person to remind you of something, using a tall building for navigating your way home, or reducing working memory load by taking notes during a conversation. Or in the case of drawing: “One draws, responds to what one has drawn, draws more, and so on. The goals for the drawing change as the drawing evolves and different effects become possible, making the whole development a mutual affair rather than a matter of one-way determinism” (Bredo, 1994, p. 28).

In philosophy, there is a debate on whether states of the body and the environment can be considered extra-neural contributors to cognition (Wilson, 2002), or in a more radical reading, external vehicles of cognition (Clark and Chalmers, 1998; Clark, 2008). According to the radical extended perspective, the internalist view is provoked by the classic thesis that “If, as we confront some task, a part of the world functions as a process which, were it to go on in the head, we would have no hesitation in accepting as part of the cognitive process, then that part of the world is (for that time) part of the cognitive process” (Clark and Chalmers, 1998, p. 8). The less radical thesis, the notion of embeddedness, also stresses a tight coupling between the agent and the world and suggests that the body and environment can, often in unexpected ways, causally impact cognition, yet suggest that the body and the environment are not part of cognition (Adams and Aizawa, 2001; Rupert, 2009). Thus the difference between embedded and extended cognition is whether extra-neural conditions causally impact cognition (embedded thesis) or are constitutive of it (extended thesis). As mentioned in the introduction, we will side-step this technical debate; for our present purposes it suffices to say that we follow the joint anti-internalist approach of embedded *and* extended cognition, which suggests that the cognitive system works in concert with the body and the environment.

The embedded/extended perspective has given rise to a large amount of empirical research on the way the cognitive system uses the body and the environment (e.g., Kirsh and Maglio, 1994; Ballard et al., 1995; Haselen et al., 2000; Martin and Schwartz, 2005; Fu, 2011; Risko et al., 2013; see also Pouw et al., 2014). A seminal study by Kirsh and Maglio (1994; see also Stull et al., 2012) found that expert Tetris players make more use of *epistemic actions*; actions that uncover (hidden) information that is cognitively demanding to compute. These types of actions are different from actions that bring one closer to one’s goal (pragmatic actions). For example, advanced players, instead of rotating “zoids” (i.e., falling block arrangements in Tetris) through mental simulation to judge whether it will fit the zoids in the bottom deck, they preferred rotating them physically as this allowed a direct matching of orientation and fit. The cognitive operation of rotation to determine a possible fit was thus off-loaded onto the environment.

Another classic study (Ballard et al., 1995, 1997; Haselen et al., 2000) showed that the cognitive system opts for retrieving information just-in-time, thereby minimizing constrains on working-memory. Participants were asked to recreate a configuration of colored blocks from a *model* by picking up colored blocks from a *resource space* and putting them in a *work-space*. The model, resource-, and work-space were all displayed in front of the participants. Eye-movement data were collected during this task. Participants made many switches of eye fixations between the model, work and -resource space. This indicated that participants adopt a “minimal memory strategy” in which information is gathered incrementally as opposed to memorized in one fell swoop. Instead of memorizing the position and color all at once, participants first memorized the color to be searched from the model, then after finding a color match in the resource space, looked up the position of the block of the model. Thus, information is gathered just in time to minimize working memory constraints (see also Cary and Carlson, 1999, who obtained similar results in an income calculation task).

Yet, findings indicate that the cognitive system does not seem to have an *a priori* preference for using the environment rather than internal cognitive resources in solving a cognitive problem; which strategy is adopted depends on the context. For example, when Ballard et al. (1995) increased the distance between the workplace and the model, participants were more likely to adopt a memory-intensive strategy. This finding resonates with the study by Gray and Fu (2004; see also Fu, 2011) in which participants were confronted with the task of programing a simulated VCR. In this task, retrieval costs of attaining task-relevant information were subtly manipulated. That is, the ease of retrieval was manipulated in such a way that participants could either acquire the information through a simple glimpse or through performing an additional mouse-click to make the information available. The cognitive strategy that the subjects chose changed as a function of the ease of retrievability. When external information was directly accessible, participants primarily relied on retrieving information externally. Attaining this “perfect-knowledge-in-the-world” was shown to be a reliable strategy, as it reduces the number of mistakes made during the task. Moreover, when the information was only indirectly available, participants were more likely to rely on internal memory, which produced a larger number of mistakes. The reason why participants in this condition relied on “imperfect-knowledge-in-the-head” was that the internally stored information was more quickly available compared to externally available information, as was predicted by a computational model that expressed the amount of time it takes to retrieve or recall information. Thus people seem to opt for the quickest problem-solving strategy in which the cognitive system “tends to recruit, on the spot, whatever mix of problem-solving resources will yield an acceptable result with a minimum of effort” (Clark, 2008, p. 13).

Situational constraints bring about a trade-off decision whether the cognitive system relies on computation performed “on-line” (with the environment) or “off-line” (internally; Wilson, 2002). Relevant in this regard is a recent set of experiments conducted by Risko et al. (2013) in which participants were presented with a varying number of letters that were either presented upright or tilted at 45° or 90°. Participants spontaneously rotated their head, which indeed seemed to promote readability of tilted presentation of letters. Furthermore, participants were more likely to rotate their head when more letters were presented and tilt of the letters was more extreme, indicating that head-tilting (which they call external normalization) occurs when the cognitive demand of not tilting the head by means of “internal normalization” increases (more cognitive effort to read more letters in tilted position, and more extreme tilt of the letters). Thus, when internal computational demand increases, an externally mediated cognitive strategy becomes more attractive. This was also found in a study by Kirsh (2009), in which participants played a mental tic-tac-toe game with the experimenter. During the mental tic-tac-toe game participants have to keep their own “moves” and those of the opponent, in mind. In the critical conditions, participants were given a sheet of paper with a tic-tac-toe matrix depicted on it or a blank sheet. External support of a tic-tac-toe matrix aided participants’ efficiency of playing the game in comparison to having no support or a white sheet. Apparently, participants are able to project the progression of the moves on the matrix through visual simulation. This is very similar to chess-players who think through moves on a chess-board without manipulating the board (Kirsh, 2009). Interestingly, however, the external support was only beneficial when the tic-tac-toe game was complex (4 × 4 matrix as opposed to a 3 × 3 matrix), and especially for participants who scored low on spatial ability. Thus, this study suggests that projection on external support is especially helpful when cognitive demand is high, and relatedly, primarily for those who are low in spatial cognitive ability.

As a final example, the study conducted by Martin and Schwartz (2005) shows how active manipulation of the environment may foster learning through exploration of the solution space. In two studies, children (9–10 years old) were learning how to solve fraction operator problems (e.g., one-fourth of eight candies), using physical tiles and pie-wedges that were movable *and* in another set of trials, using line drawings of pies or tiles which they could highlight and circle with a pen. The difficulty that children often experience in this task is that they focus on the numerator, leading them to understand “one-fourth of eight candies” to be “one candy.” Martin and Schwartz (2005) predicted that physical interaction with manipulable objects would increase the chance that children come to interpret that one-fourth of eight means four groups of two because rearranging the tiles results in new groupings. Thus they reasoned that the agent and the environment mutually adapt each other (as in the case of drawing), where one acts without a preconceived goal on the environment which in turn feeds back information that might align with the correct solution. Indeed, children performed better with manipulable objects than without them (Experiments 1 and 2). Interestingly, presenting the children with the correct organization of tiles did not aid understanding; rather the physical open-ended interaction with the environment drove understanding and performance on the task (see also Manches et al., 2010).

Let us summarize. First, the cognitive system makes use of the environment to distribute computational load but also to enable exploration of a problem-space that is difficult to achieve off-line (i.e., to achieve through purely internal computations). Moreover, the cognitive system is not *a priori* driven to reduce internal computational load by off-loading onto the environment, rather the environment is exploited if it offers a cheaper resource than internal means of computation to achieve an acceptable performance on a task (Gray and Fu, 2004). Although not conclusive, it further seems that when cognitive demand is high, either due to external constraints (higher cognitive load of the task) or internal constraints (e.g., low visuospatial cognitive ability) the cognitive system is more likely to opt for and benefit from external computational strategies. However, these findings do not allow us to draw definitive conclusions about when and how the cognitive system trades external with internal computational resources. Thus one of the major challenges for research in embedded/extended cognition is to determine which external (e.g., availability of external information) and internal (e.g., working memory ability) constraints affect whether and how problem-solving strategies become externally or internally mediated (Risko et al., 2013). Furthermore, is it possible to identify a trajectory of problem-solving strategies as expertise develops? Specifically, does the cognitive system first rely on external support – given that it is still ill-equipped to perform stand-alone internal computations – and are computations increasingly performed off-line when the cognitive system becomes more equipped (e.g., because of acquired strategy knowledge or chunking mechanisms) to hold task-relevant information internally?

Even though such questions cannot yet be answered by the embedded/extended cognition frameworks, it is not difficult to see the relevance of this framework for gesture research; there is a clear analogy between these findings and the findings from some of the gesture studies reviewed in the section on “the intra-cognitive role of gestures.”

### AN EMBEDDED/EXTENDED PERSPECTIVE ON THE COGNITIVE FUNCTION OF GESTURES

Recently, Clark (2008, 2013; see also Wheeler, 2013) provided a purely extended perspective on gesticulation. Clark (2013) provides a detailed discussion of why gestures should be seen as constitutive to – as opposed to merely causally impinging on – cognitive processes (cf. Wheeler, 2013). Here we only briefly address his account to further develop an embedded/extended perspective that is able to provide an explanation of the empirical data on the cognitive function of gestures as well as produce hypotheses and identify challenges for further research.

According to Clark (2013) we should *not* understand the cognitive role of gestures purely in terms of its neural pre- and post-cursors:

“The wrong image here is that of a central reasoning engine that merely uses gesture to clothe or materialize performed ideas. Instead, gesture and overt or covert speech emerge as interacting parts of a distributed cognitive engine, participating in cognitively potent self-stimulating loops whose activity is as much an *aspect* of our thinking as its *result.*” (p. 263)

Furthermore, he states that:

“The physical act of gesturing is part and parcel of a coupled neural-bodily unfolding that is itself usefully seen as an extended process of thought.” (p. 257)

Clark further argues that by producing a gesture, something concrete is brought into being (arm posture) that subsequently affects ongoing thinking and reasoning. Much like using a notepad, gestures provide a *stable* physical presence that embodies a particular aspect of a cognitive task. We can appreciate Clark’s point if we consider that speech dissolves in midair and working memory allows only for a certain amount of thoughts to be consciously entertained. We can argue that gestures are not only a way to externalize speech and thought content, but also allow for temporal cognitive stability that might be more reliable than internal means of temporal cognitive extension (e.g., consciously attending to a thought to keep in mind).

Thus the key to an embedded/extended perspective on gestures is the view that gestures fulfill a cognitive function *because* they are bodily. That is, in contrast to what the GSA and the IPH propose, gesticulation produces an external physical presence that somehow supports internal cognitive processes. According to Clark’s (2013) purely extended account, this physical presence instantiated in gesture is actually part of thinking itself. Indeed, he thinks that a more moderate account of gestures’ function in which they merely *affect* inner neural cognitive processes is misconstrued. His argument for an extended cognitive understanding of gestures relies on the appreciation that some crucial forms of neural activity arise in coordination with gestures, wherein gesture and neural activity are interdependent in achieving a particular cognitive state. Thus although, in some instances “‘neural goings-on’ may be sufficient for the presence of some cognitive state or the other” in other instances gestures, at times, should be given a genuine cognitive status (p. 261) because “gesture and speech emerge as interacting parts of a cognitive system” (p. 263) whereby no meaningful categorization can be made of what should be considered cognitive or non-cognitive on the basis of the distinction between inner (neural activity) and outer (gestures).

How and when do these specific physical conditions fulfill a supporting role for a particular cognitive function? It is instructive to compare the research from the embedded/extended cognition tradition with research on the cognitive function of gesture. We need to reconsider the research by Kirsh and Maglio (1994), which showed that expert Tetris players operate on the environment to alleviate internal computational load (epistemic actions). Determining where a zoid fits is not dependent on internally computed rotations of the zoid, but is achieved by actual rotation of the zoid. In mental rotation tasks in which participants have to judge whether a 3-d zoid matches one out of several 3-d zoids depicted in different rotational angles (classic S–M cube task; Shepard and Metzler, 1971), participants use gestures to aid in their judgments (Chu and Kita, 2008, 2011). We would submit, that gestures in this case *are* epistemic actions that reveal information that is hidden (since the 3-d zoids do not rotate by themselves) and difficult or more costly to compute internally. Chu and Kita (2008) also found that when participants first approach the mental rotation task they are more likely to use hand-movements *as-if* actively rotating the block. We would speculate that in this case gestures fulfill the function of providing a physical platform that supports the internal representational stability (a term earlier used by Hutchins, 2005) of a rotating 3-d zoid (see also Pouw et al., 2014). In this case the zoid is visually “projected” into the hands (Kirsh, 2009) and is manipulated *as if* it were actually in the hand. In this case the hands offer a reliable external support for performing the cognitive function of rotating the projected 3-d zoid through gestures. Furthermore, using pointing gestures to keep track of something in the environment similarly produces a reliable physical attentional marker that alleviates internal attentional tracking processes (e.g., Kirsh, 1995; Delgado et al., 2011). This might also be the case with abacus users doing mental calculations that perform gestures on, what seems to be, a mentally projected abacus (Hatano et al., 1977; Hatano and Osawa, 1983). In this case, physical gesticulation seems to be preferred by these users as opposed to internally simulating changes on the abacus. We would argue that because gestures allow a stable external physical presence, they support internal representational stability of the dynamically changing abacus during calculation. In line with Kirsh (2009), we argue that in these cases the cognitive system seems to be neither purely off-line nor on-line; rather, it uses partly environmental resources (e.g., gestures) and internal cognitive resources (e.g., visual simulation) to perform a task. Gestures are essentially a way to put on-line extra-neural resources into the mix of problem-solving resources.

Another possible embedded/extended function of gesture is exploration of a problem space. Martin and Schwartz (2005) found that manipulation of objects promoted the understanding of fraction-operating principles. Relevantly, gesturing might sometimes allow the gesturer to become aware of structural correlations that would be difficult to generate through internal computation. For instance, this seemed to be the case in the rotating-gear problem, in which the number gestures used that simulated each rotation of a gear predicted the discovery of a more efficient problem-solving strategy that involved pick-up of the regularity that each gear *N* + 2 rotates in the same direction (Delgado et al., 2011).

With regard to when gestures emerge to fulfill an embedded/extended function, the research that we have discussed in the domain of embedded/extended cognition has another interesting alignment with the gesture literature. We can summarize both streams of findings in one converging main principle: *When the costs of internal computation are high, either induced by external constraints (higher cognitive demand of the task; more cost of retrieving information from the environment) or internal constraints (e.g., lower working memory ability) the cognitive system is more likely to adopt, if cheaply available, an externally supported problem-solving strategy; be it the environment or gestures* (Goldin-Meadow et al., 2001; Gray and Fu, 2004; Wagner et al., 2004; Kirsh, 2009; Ping and Goldin-Meadow, 2010; Marstaller and Burianová, 2013; Risko et al., 2013; Smithson and Nicoladis, 2014). In other words, “cognitive processes flow to wherever it is cheaper to perform them” (Kirsh, 2010, p. 442). Understood in this manner, it is not surprising that people who are describing a physical object tend to gesture less when the object is present as opposed to absent (Morsella and Krauss, 2004), since the task-relevant information is cheaply available in the environment. Or that gestures are more likely to be used to lighten the cognitive load when pressure is put on internal computational system (cognitive demand of the task; e.g., Goldin-Meadow et al., 2001; Smithson and Nicoladis, 2014).

This embedded/extended perspective on the cognitive function of gestures, leads to several testable questions and further challenges for future research.

First, an interesting avenue for further research is to determine how changes in the external constraints – such as the cognitive demands of a task – and in the ease of availability of external resources, changes the likelihood of gesturing. For example, one could devise a mental rotation task in which participants can rotate a 3-d zoid either through a mouse, by using gestures, or solely by internal strategies. According to the present perspective, if we manipulate the speed in which the 3-d zoid can be manipulated by a mouse, we would predict that participants are more likely to use gestures when the manipulation takes more time (as relative cost decreases). Another, more unorthodox manipulation would be to put varying weights on the wrists of participants, which may induce costs in terms of energy expense, leading participants to an earlier adoption of an internal solution strategy. Many more constraints could be considered to assess the trade-off decision between internal and external resources that the cognitive system seems to make.

Second, gesture use evolves (Chu and Kita, 2008). When the task is more familiar, hand-gestures evolve from “*as-if* manipulations” to a stand-in-for relation of the 3-d zoid by means of a rotating flat hand, eventually eliminating the use of gestures altogether. In a similar vein, when abacus users become more advanced they tend to use less and less gestures during mental calculations. Indeed, it seems that gestures itself are costly to perform, and contrary to the GSA account, may under certain circumstances hinder performance (De Nooijer et al., in press), or learning (Post et al., 2013) relative to other strategies. Interesting in this regard, is research that suggests that different types of body-movements have their own cognitive load (or come with particular cognitive costs) and may at times be traded for less costly bodily movements. That is dancers who rehearsed a dance-routine performed better when they rehearsed through “marking” (minimal movements and use of gestures to stand in for full-out movements) as opposed to rehearsing the routine full out (Warburton et al., 2013). Thus, it seems that under certain conditions, gestures, once cheap resources to think with, become relatively costly in comparison to, and are therefore traded in for, purely internal strategies. This raises several questions. For example, do gestures help in the internalization process? Thus, are embedded/extended solution strategies shaping the way internal computations are performed?

Relatedly, when the cognitive system has a lower ability to produce internal object rotations (i.e., low spatial cognitive ability) it will rely more on external resources such as gestures (e.g., Chu et al., 2013; Marstaller and Burianová, 2013). An important research question that relates to this idea is whether people who score “low” on spatial cognitive ability test are actually only scoring low on *mental* spatial cognitive ability, and may not underperform when gestures are allowed. Indeed, when gesture is prohibited people who are low in working memory perform only more poorly on a mental rotation task with no performance deficits in the gesture condition, suggesting that they can fully compensate with external problem-solving strategies (Marstaller and Burianová, 2013). Furthermore, consider findings that prohibiting gesturing has a negative effect on performance. Seen in this light, this negative effect of not gesturing may not arise because it imposes cognitive load, and thereby imposes constraints on cognition (as proposed by the GSA account), but precisely because the prohibition to gesture withholds the cognitive system from the use of external resources in the performance of a task. Thus, whereas the GSA account suggests that not-gesturing imposes a cognitive load since the agent has to prevent automatic activations of gestures, we propose that the prohibition of gesturing takes external bodily resources away from the agent and drives the agent to rely exclusively on internal computational processes. This is an important empirical question that future research should address, as it is both related to how we should define and measure cognitive abilities, as well as to the particular cognitive function of gestures.

A more fundamental question that currently remains unanswered in the embedded/extended perspective on gesturing is what type of information is being made available through gesturing. Is it the proprioceptive, kinesthetic, haptic, and/or visual consequences of movement that allow gestures to support cognitive processes? Or both, as these systems are tightly coupled (e.g., Radman, 2013)? For example, it is well-known that the visually impaired people use gestures (Iverson, 1998). Do they still benefit from gestures through proprioception or other consequences of movement? Clark (2013) raised a similar question in relation to patients with a rare disease that leads to loss of proprioception; yet these patients are still able to gesture quite naturally (see Gallagher, 2005).Would gestures still fulfill an embedded/extended cognitive function for such patients through visual feedback? This question is somewhat harder to address since the disease is, luckily, quite rare. An interesting avenue for research therefore would be to interfere with the information that gestures might provide as to identify factors that might underlie the embedded/extended cognitive function of gestures. For example, obstructing visibility of one’s own gestures, by putting a screen at the level of the shoulders (Gallagher, 2005). Thus the current challenge for the present account is to provide an account of what information gestures produce that might be supportive for cognitive processes.

## CONCLUSION

By means of our review of the empirical literature we have tried to assess explanatory power of current theories with regard to the question of how gestures might fulfill cognitive functions. Although all the accounts we have addressed here claim that gestures indeed fulfill a cognitive function, we have shown that in these accounts, this claim often does not refer to gestures, but rather to their neural precursors. Importantly, there are accounts that suggest that gestures fulfill the cognitive role of priming or activating internal action representations (e.g., Krauss et al., 2000; Goldin-Meadow and Beilock, 2010), yet we think the reason why bodily movements fulfill this function is not clearly stated and seems to differ from the embedded/extended cognitive function we have identified here. We have tried to analyze the cognitive functions of gestures, by integrating the literature of embedded/extended cognition with the gesture literature. There is a considerable amount of overlap between the ways cognizers have been found to use their environment as well as how gestures support cognitive processes. Although further research into the exact mechanisms of embedded/extended functions of gestures is necessary, we put forth the notion that gestures provide the cognitive system with a stable external, physical, and visual presence that can provide a means to think with.

Importantly, we should stress two related concerns that apply to the current proposal. First, it is evident that the embedded/extended view on gestures, as presented here, does not address the full gamut of gesticulation. We have primarily focused on co-thought gestures in problem-solving contexts instead of, for example, beat gestures, or gestures that primarily emerge in communicative contexts. Therefore, at this point we remain agnostic to whether all gestures fulfill an embedded/extended cognitive function (for the gesturer). Indeed, extant “alternative” theories that we have addressed here may very well be complementary to our proposal. These theories are complementary to our proposal in that they might address cognitive functions and underpinnings of gestures that we have not addressed here. For example, it is possible that gestures emerge from action-related motor simulations that are activated during visuo-spatial cognition (Hostetter and Alibali, 2008) with the added proposal that the bodily externalizations of these motor simulations have a cognitive function themselves of the kind we have proposed here. Thus although we maintain that current theories in the gesture literature are not very suitable to address why gestures-as-bodily-acts might fulfill a cognitive function, our proposal does not deny any explanatory power of these theories regarding other aspects of the nature and cognitive function of gestures.

Secondly, it is clear that gestures have a developmental trajectory and primarily emerge in intersubjective contexts (e.g., McNeill, 1992; Iverson and Thelen, 1999; Tomasello, 2008; Liszkowski et al., 2012). As such, the current embedded/extended account of the cognitive function of gestures is still presented in an “ontogenetic vacuum” *and* is still rather individualistic. Although this is a concern that needs to be addressed in future work, there is much room for exploring how the embedded/extended function of gestures might be related to developmental and social dimensions. For example, Iverson and Thelen (1999) have provided a detailed account of how the hands, mouth, and the brain should be regarded as one dynamical system; more specifically of how these components become entrained throughout development. Although they focus primarily on the way language and gesture become constitutively interdependent, the kind of gestures that have been the focus of this paper (gestures in problem-solving contexts) can be scaffolded onto their developmental account as another way of how “perception, action, and cognition can be mutually and flexibly coupled” (Iverson and Thelen, 1999, p. 37). On the other hand, how does our account relate to the intersubjective context in which gestures most often emerge? It would fare well with appeals coming from embodied cognitive science which suggest that an important way humans achieve interpersonal understanding is not from a spectatorial third-person stance, but rather from an interactive and second-person stance (e.g., De Jaegher and Di Paolo, 2007; De Jaegher et al., 2010; Anderson et al., 2012; Schilbach et al., 2013; Pouw et al., under review). In these approaches interpersonal understanding involves “know-how that allows us to sustain interactions, form relations, understand each other, and act together” (De Jaegher et al., 2010, p. 442), instead of two brains trying to predict each other’s mental contents through observation alone. In such a portrayal of intersubjectivity, gestures are always already considered as having an embedded function for both the gesturer and the interlocutor since gestures are co-constitutive of the social coordination itself. To put it another way, in social interaction gestures are a non-neural component that is part of an organism–organism–environment coordinative structure (Anderson et al., 2012). The challenge for further work is to show how non-social embedded/extended gestures that we have focused on here might develop from these social contexts.

In closing, our aim with this article to point out the necessity of understanding the role of the body in thinking. We tried to accomplish this by developing an embedded/extended perspective on the cognitive role of gestures. In this perspective, the body is not a trivial output-appendage of the cognitive system but an important component thereof. The body is a resource with particular qualities that is recruited in the coordination of cognitive processes. This perspective intended to promote research that tries to further address when, why, and how gestures are recruited during cognitive processes.

## AUTHOR CONTRIBUTIONS

Wim T. J. L. Pouw drafted, Jacqueline A. de Nooijer co-drafted, Tamara van Gog, Rolf A. Zwaan, and Fred Paas provided critical revision of the manuscript. All authors approved the final manuscript.

## Conflict of Interest Statement

The authors declare that the research was conducted in the absence of any commercial or financial relationships that could be construed as a potential conflict of interest.

## Footnotes

1 Defined as “iconic depictions of abstract concepts of meaning and language” (McNeill, 1985, p. 357).
